# Impact of Lignite Combustion Air Pollution on Acute Coronary Syndrome and Atrial Fibrillation Incidence in Western Macedonia, Greece

**DOI:** 10.3390/ijerph23010113

**Published:** 2026-01-16

**Authors:** Vasileios Vasilakopoulos, Ioannis Kanonidis, Christina-Ioanna Papadopoulou, George Fragulis, Stergios Ganatsios

**Affiliations:** 1 Department of Electrical and Computer Engineering, University of Western Macedonia, 50100 Kozani, Greece; 2 2nd Department of Cardiology, Hippokrateio General Hospital, Aristotle University Medical School, 54124 Thessaloniki, Greece; 3 General Hospital of Ptolemaida, 50200 Ptolemaida, Greece

**Keywords:** air pollution, particulate matter, lignite, acute coronary syndrome, atrial fibrillation, cardiovascular risk, Western Macedonia

## Abstract

**Highlights:**

**Public health relevance—How does this work relate to a public health issue?**

- The study demonstrates that chronic exposure to PM_10_, SO_2_ and NO_x_ from lignite combustion significantly increases population-level cardiovascular risk, particularly for Acute Coronary Syndromes (ACS) and Atrial Fibrillation (AF).
- By analyzing real-world data from a coal-dependent region, it shows how air pollution acts as a direct and measurable determinant of acute cardiac events at the community level.

**Public health significance—Why is this work of significance to public health?**

- The sharp decline in pollutant levels after the lignite phase-out was accompanied by a large reduction in ACS and AF admissions, revealing that air quality improvements translate rapidly into improved cardiovascular outcomes.
- The findings provide robust regional evidence that emission-reduction policies function as effective cardiovascular prevention strategies, complementing clinical and behavioral interventions.

**Public health implications—What are the key implications or messages for practitioners, policy makers and/or researchers in public health?**

- Policymakers should treat air quality management and fossil-fuel phase-out as essential public health interventions, with substantial potential to reduce cardiac morbidity and healthcare burden.
- Clinicians and public health authorities should incorporate air pollution exposure into cardiovascular risk communication and develop targeted advisories for vulnerable populations during high-pollution periods.

**Abstract:**

Air pollution from lignite combustion represents a major environmental and public health concern, particularly for cardiovascular disease. This study investigated the relationship between ambient air pollution and hospital admissions for Acute Coronary Syndromes (ACS) and Atrial Fibrillation (AF) in Western Macedonia, Greece—a region historically dominated by lignite mining and power generation. Air quality data for PM_10_, SO_2_, and NO_x_ from 2011–2014 and 2021 were analyzed alongside hospital admission records from four regional hospitals (Kozani, Ptolemaida, Florina, Grevena). Spatial analyses revealed significantly higher pollutant concentrations and cardiovascular admissions in high-exposure areas near power plants compared with the control area. Temporal analyses demonstrated a pronounced decline in pollutant levels between 2014 and 2021, coinciding with lignite phase-out and accompanied by a marked reduction in ACS and AF hospitalizations, particularly in the high-exposure areas of Ptolemaida and Florina. Correlation analyses indicated modest but significant positive associations between monthly pollutant concentrations and cardiovascular admissions. These findings provide real-world evidence that reductions in air pollution following lignite decommissioning were associated with improved cardiovascular outcomes. The study underscores the medical importance of air quality improvement and highlights emission reduction as a critical strategy for cardiovascular disease prevention in transitioning energy regions.

## 1. Introduction

Air pollution represents a pervasive environmental health hazard with substantial cardiovascular implications. On a global scale, ambient air pollution—primarily fine particulates and gases resulting from fuel combustion—accounts for an estimated 6–7 million premature deaths each year and ranks as the fourth leading risk factor for mortality worldwide [1,2]. Cardiovascular causes constitute more than two-thirds of these pollution-attributable deaths, particularly ischemic heart disease and stroke [2]. In Europe, emissions from coal-fired power plants alone are estimated to cause approximately 33,900 excess deaths annually due to cardiovascular and respiratory diseases [3].

Among the pollutants of greatest relevance to cardiovascular health are particulate matter and toxic gases generated by fossil fuel combustion. Particulate matter ≤ 10 μm in diameter (PM_10_) and fine particles ≤ 2.5 μm (PM_2.5_) are capable of penetrating deep into the lungs and systemic circulation, provoking systemic inflammation, oxidative stress, endothelial dysfunction, and coagulation abnormalities [4,5]. These mechanisms are known to precipitate acute cardiovascular events and exacerbate chronic disease. Epidemiological studies have demonstrated that even short-term elevations in particulate concentrations can trigger acute coronary syndromes (ACS), encompassing myocardial infarction (MI) and unstable angina. A landmark meta-analysis by Mustafic et al. reported that all major air pollutants except ozone were significantly associated with a near-term increase in myocardial infarction risk [6]. For a 10 μg/m^3^ increase in pollutant concentration, relative risks in the range of 1.01–1.03 have been documented for acute MI occurrence [6,7]. Long-term exposure appears even more detrimental; each 5–10 μg/m^3^ rise in fine PM_2.5_ concentration has been associated with several-percent increases in coronary mortality and atherosclerotic cardiovascular events over time [7,8]. Importantly, pollution-related cardiovascular risk persists even at concentrations below existing regulatory thresholds [9], suggesting that no exposure level can be considered entirely safe.

Air pollution also plays a significant role in the pathogenesis of cardiac arrhythmias. Atrial fibrillation (AF)—the most common sustained cardiac arrhythmia—has increasingly been associated with environmental exposures such as particulate and gaseous pollutants [10,11]. Mechanistically, pollution may contribute to AF through autonomic nervous system imbalance, oxidative injury to cardiomyocytes, and enhanced systemic inflammation, which collectively foster atrial remodeling and electrical instability [5,12]. A growing body of epidemiological research reinforces this association. Recent meta-analyses indicate that both short-term pollution spikes and chronic exposure are correlated with elevated AF incidence. Chen et al. [9] analyzed 18 studies and concluded that acute exposure to PM_2.5_, NO_2_, and SO_2_ significantly heightens the immediate risk of AF episodes, whereas chronic exposure to pollutants (PM_2.5_, PM_10_, NO_2_, SO_2_, CO) increases the long-term incidence of AF among healthy populations [13,14]. Similarly, Yue et al. [10] reported that each 10 μg/m^3^ rise in daily PM_10_ or PM_2.5_ levels corresponds to a 1–3% increase in AF onset odds, while prolonged exposure to fine PM elevates AF prevalence by approximately 7% per 10 μg/m^3^ increment [15,16]. An umbrella review encompassing 16 observational studies found a pooled 11% increase in AF risk per 10 μg/m^3^ increment in PM_2.5_, with more pronounced effects observed in regions with heavy pollution burdens [17,18]. Although these relative risks appear modest, their public health impact is substantial, given the ubiquity of pollution exposure and the high baseline prevalence of AF [10,19]. Collectively, the evidence supports the recognition of air pollution as a modifiable cardiovascular risk factor—comparable in importance to traditional factors—and justifies the inclusion of pollution mitigation in preventive cardiology strategies [20,21].

Western Macedonia, Greece, constitutes a distinctive real-world context for examining the relationship between air pollution and cardiovascular health. Located in the country’s northwest, the region has historically been recognized as Greece’s “lignite heartland.” Throughout the latter half of the 20th century and into the early 2000s, Western Macedonia hosted extensive lignite (brown coal) mining and several large coal-fired power stations operated by the Public Power Corporation (PPC) [22,23,24]. At its peak, the Western Macedonia Lignite Center comprised six coal power plants with a combined installed capacity of approximately 4.4 GW, supplying a major portion of Greece’s electricity [22]. Concentrated around the Ptolemaida–Kozani basin and the Florina area, these operations emitted substantial quantities of PM, SO_2_, NO_x_ (NO + NO_2_), and other pollutants over several decades, leading to sustained air quality degradation [25,26]. Local investigations have documented elevated particulate concentrations and heavy metal content in ambient dust, as well as adverse respiratory outcomes among residents living near mining and power plant sites [27,28]. For example, schoolchildren in the heavily polluted town of Ptolemaida exhibited significantly higher rates of chronic bronchitis and rhinitis compared with peers in the relatively cleaner and non-industrialized town of Grevena [28]. However, the cardiovascular consequences for the regional population have not been comprehensively quantified in the existing literature. Given that air pollution levels in Western Macedonia have historically exceeded national averages and frequently surpassed European Union air quality limits [29,30], a corresponding burden of pollution-related cardiac events would be anticipated.

In recent years, Greece has embarked on an ambitious energy transition, implementing policies to phase out lignite combustion in alignment with climate and air quality objectives. Lignite production in Western Macedonia has decreased sharply since the early 2010s, from a peak of approximately 70 million tons in 2002 to around 10 million tons by 2020 [31]. Several older power plant units were decommissioned or curtailed after 2014, and a complete phase-out of coal-fired generation is targeted by 2028. This process provides a natural quasi-experimental framework for assessing whether air quality improvements correspond with better cardiovascular health outcomes. The present study hypothesizes that exposure to air pollution from PPC’s lignite power plants is associated with higher rates of acute cardiac events—specifically acute coronary syndromes and atrial fibrillation—among local populations, and that recent reductions in pollution will coincide with a decline in these events.

Accordingly, this study investigates the relationship between ambient air pollution (predominantly from lignite combustion emissions) and hospital admissions for cardiovascular conditions in Western Macedonia. The analysis focuses on Acute Coronary Syndromes and Atrial Fibrillation as two clinically significant outcomes potentially linked to pollution exposure. Environmental monitoring data are integrated with hospital records from multiple regional healthcare facilities over an extended time frame. By analyzing both spatial variations (high- versus low-pollution areas) and temporal trends (before versus after emission reductions), the study seeks to elucidate the impact of lignite-related air pollution on ACS and AF incidence. The findings address a critical gap in regional environmental health research and offer insights relevant to other coal-dependent regions globally. Ultimately, the results underscore the medical importance of air quality improvement and reinforce air pollution mitigation as a strategic approach to cardiovascular disease prevention.

While the adverse cardiovascular effects of air pollution are well established, empirical evidence capturing health responses to large-scale reductions in coal-related emissions remains limited. The present study contributes novel real-world evidence by exploiting the lignite phase-out in Western Macedonia as a natural experiment, linking source-specific emission reductions to changes in acute cardiovascular outcomes across an entire regional healthcare system.

## 2. Materials and Methods

### 2.1. Study Area and Population

**Figure 1:**
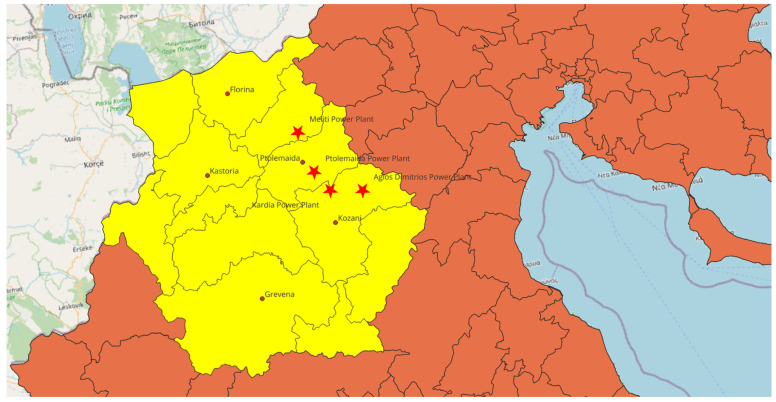
Administrative map of Western Macedonia (Greece) showing the study area, the main urban centers (Kozani, Ptolemaida, Florina, Grevena), and the location of PPC lignite-fired power plants. Source: author’s own creation.

The investigation was conducted in Western Macedonia, an administrative region located in northwestern Greece that encompasses the regional units of Kozani, Florina, Grevena, and Kastoria. The region has a population of approximately 283,000 residents and is characterized by a continental Mediterranean climate with cold winters and warm, dry summers [32]. Historically, Western Macedonia has been dominated by lignite mining and electricity production [33]. The principal lignite basin extends across the Kozani–Ptolemaida plain and into Florina, where multiple surface mines and coal-fired power stations have operated for several decades. The main power plants functioning during the study period included Ptolemaida, Agios Dimitrios, Kardia and Meliti, all of which utilized locally extracted lignite. The municipalities of Kozani and Ptolemaida lie at the center of the mining and power-generation complex, whereas Florina is situated in a northern sub-basin containing its own lignite plant (Meliti). In contrast, Grevena, located to the west, lacks significant industrial sources and serves as a rural background reference area. Figure 1 presents the administrative map of Western Macedonia (Greece), clearly identifying the studied regional units and major urban centers (Kozani, Ptolemaida, Florina, Grevena), as well as the locations of PPC lignite-fired power plants. Importantly, lignite has not been used for domestic heating in the study area, as its household use is prohibited in Greece. Residential heating in Kozani, Ptolemaida, and Amyntaio is predominantly supplied through district heating systems, while Grevena lacks both district heating and lignite-based domestic fuel use. Consequently, domestic lignite combustion does not constitute a confounding source of particulate or gaseous emissions in any of the study communities.

For health data collection, four study communities were defined based on the catchment areas of the regional general hospitals located in Kozani (the regional capital), Ptolemaida (an industrial town within the Kozani unit), Florina (a town near the northern lignite facility), and Grevena (a smaller town situated in a non-industrial area). These hospitals constitute the primary public healthcare facilities serving the majority of the local population [34]. The population sizes within the respective hospital catchment areas during the study period were estimated at approximately 150,000 for Kozani and Ptolemaida combined (as they are part of the same broader area), around 50,000 for Florina, and approximately 30,000 for Grevena. Ethical approval for the retrospective analysis of de-identified hospital admission data was obtained from the review boards of all participating institutions, and the study was carried out in accordance with the principles of the Declaration of Helsinki.

### 2.2. Air Pollution Data

Ambient air quality data were obtained from the Western Macedonia regional monitoring network, operated by the University of Western Macedonia. Monitoring stations situated near the four hospital communities were selected to represent local exposure levels. These included stations located in the cities of Kozani and Florina, one near Ptolemaida (adjacent to the lignite mines), and a background station in Grevena. The precise geographic coordinates (latitude and longitude) of all air quality monitoring stations are reported in Table 1. The analysis focused on key pollutants emitted by lignite combustion and known to be relevant to cardiovascular health: particulate matter with aerodynamic diameter ≤ 10 μm (PM_10_), sulfur dioxide (SO_2_), nitrogen monoxide (NO) and nitrogen dioxide (NO_2_)—collectively expressed as nitrogen oxides (NO_x_)—as well as ozone (O_3_) for contextual comparison. Continuous monitoring data were retrieved for the years 2011, 2012, 2013, 2014, and 2021, providing complete datasets for all selected stations. These years represent two distinct time periods corresponding to the pre- and post-intervention phases. Owing to technical issues and network reconfiguration, uniform data coverage for 2015–2020 was not available. Therefore, comparisons were performed between the years 2011–2014, when all power plants were fully operational, and 2021, when several units had been shut down or had significantly reduced operation. The year 2021 was selected as the post-intervention reference because it represents the first year with substantially reduced lignite activity and complete, comparable air quality data across all monitoring stations, whereas the period 2015–2020 was characterized by incomplete data coverage and the confounding effects of the COVID-19 pandemic. All air pollution measurements were derived from officially operated fixed monitoring stations subject to routine calibration, maintenance, and quality control procedures implemented by the responsible environmental authorities. PM_2.5_ data were not included in the analysis because such measurements were not consistently available across all monitoring stations and years, and their inclusion would have compromised temporal and spatial comparability. After 2021, most lignite-fired power plants in Western Macedonia were permanently shut down, and the regional air quality monitoring network underwent restructuring, resulting in the absence of complete and homogeneous data across all study sites. Consequently, subsequent years were not included in order to preserve temporal and spatial comparability.

For each pollutant and monitoring site, monthly mean concentrations (for PM_10_, NO, NO_2_, NO_x_, and O_3_) and monthly cumulative values for SO_2_ (to capture episodic peak behavior) were computed. Monthly aggregation was employed to reduce short-term variability and to reflect the duration over which subacute health effects typically manifest. These monthly pollutant metrics were subsequently merged with monthly hospital admission counts for correlation analysis. Annual mean concentrations for each pollutant were also calculated to enable comparison between the early 2010s and 2021, thus allowing assessment of long-term air quality trends.

Quality control procedures were applied by excluding any monthly datasets with more than 25% missing daily values. Overall data capture exceeded 90% for all station–year combinations included in the analysis. Monitoring and measurement methods adhered to European Union reference standards, including gravimetric or β-attenuation techniques for PM_10_, ultraviolet fluorescence for SO_2_, and chemiluminescence for NO_x_, ensuring comparability and reliability across both spatial and temporal dimensions.

### 2.3. Hospital Admission Data

Hospital admission data were collected from four general hospitals in the study area: The geographic coordinates of the hospitals are provided in Table 1 to ensure transparency regarding their spatial location within the study area. Kozani “Mamatsio” General Hospital, Ptolemaida “Bodosakeio” General Hospital, Florina General Hospital, and Grevena General Hospital. Records were obtained for the years 2011, 2012, 2013, 2014, and 2021 to align with the available air quality data. From each hospital archive, the monthly number of cardiovascular-related admissions was retrieved. For consistency, cardiovascular admissions were defined as cases with primary diagnoses corresponding to ischemic heart disease, cardiac arrhythmias, heart failure, cerebrovascular disease, or other cardiac conditions, based on ICD-10 codes I20–I25, I48, I50, I60–I69, and related categories [35,36]. All cardiovascular diagnoses were coded using standardized ICD-10 classification criteria, which remained unchanged throughout the study period, ensuring consistency and comparability of diagnostic practices across hospitals and over time.

The monthly admissions were further classified into two principal subgroups of clinical interest: Acute Coronary Syndrome (ACS) and Atrial Fibrillation (AF). ACS cases were identified through diagnostic codes corresponding to acute myocardial infarction and unstable angina (ICD-10 I21, I22, I20.0), representing acute ischemic cardiac events [37]. AF admissions were identified using ICD-10 codes I48.x, which include atrial fibrillation and atrial flutter, typically corresponding to hospitalizations for AF with rapid ventricular response, initiation of antiarrhythmic therapy, or AF-related complications such as stroke prevention and management [36]. Other cardiac conditions (e.g., stable angina, valvular heart disease, hypertensive crises) were grouped together for completeness but were not analyzed separately. Monthly totals for overall cardiovascular admissions, ACS cases, and AF cases were compiled for each hospital and used in subsequent analyses. To account for differences in population size and hospital catchment areas, ACS and AF admissions were analyzed and presented primarily as proportions of total cardiovascular hospital admissions, enabling standardized comparisons across hospitals and over time.

Air pollutant concentrations were analyzed on a monthly basis and summarized annually for descriptive purposes. For each monitoring station and pollutant, annual statistics were calculated based on approximately 12 monthly observations per year, resulting in a total of approximately 60 data points per station over the study period. The number of observations contributing to each annual summary is reported alongside the corresponding results.

### 2.4. Statistical Analysis

Both spatial and temporal analyses were conducted to examine associations between air pollution and cardiovascular hospital admissions. Spatial comparisons were first performed to assess differences in pollutant exposure across the four communities. One-way ANOVA or Kruskal–Wallis tests (depending on data normality) were applied to annual mean pollutant concentrations [38,39,40] among the four monitoring locations (Kozani, Ptolemaida, Florina, and Grevena). Post hoc pairwise comparisons with Bonferroni correction were used to identify statistically significant differences between sites. Similarly, annual mean numbers of cardiovascular admissions were compared across the four hospitals using Chi-square tests for homogeneity, adjusting for population size differences [41]. These analyses aimed to determine whether communities exposed to higher pollution levels (Ptolemaida and Florina) experienced a greater burden of cardiac hospitalizations compared with lower-exposure areas (Grevena).

Temporal trend analyses were subsequently undertaken to evaluate changes between the 2011–2014 period and 2021. Year-by-year comparisons of pollutant concentrations were conducted using ANOVA or Kruskal–Wallis tests for each community [42]. Chi-square tests were also employed to detect differences in the distribution of cardiac admission diagnoses over time within each hospital. In particular, changes in the proportion of admissions attributed to ACS or AF were examined to determine potential shifts in cardiovascular risk patterns over the decade.

To assess direct associations between pollution levels and hospitalizations, non-parametric correlation analyses were performed. Spearman’s rank correlation coefficients (ρ) were calculated between monthly pollutant concentrations and monthly hospital admission counts for each hospital and pollutant. Given the relatively small number of monthly observations (*N* = 60 per hospital) and potential non-linear relationships, Spearman’s ρ was selected in preference to Pearson’s correlation. Correlations were tested for key pollutant–outcome pairs (e.g., PM_10_ versus total cardiac admissions, NO_2_ versus admissions), with particular emphasis on ACS and AF admissions when sample sizes permitted. A two-tailed *p*-value below 0.05 was considered statistically significant. These analyses were exploratory in nature and aimed to identify concurrent short-term variations between air pollution levels and cardiovascular hospitalization rates.

All statistical analyses were performed using SPSS version 26 and R software version 4.1.2. Results are reported together with their corresponding test statistics (χ^2^, ρ) and 95% confidence intervals where applicable.

## 3. Results

### 3.1. Air Pollution Levels in High- vs. Low-Exposure Areas

Air quality in Western Macedonia varied substantially by location, reflecting the influence of lignite-related activities. Table 2 presents mean concentrations of major pollutants in the three lignite-affected areas (Kozani, Ptolemaida, Florina) versus the cleaner control area (Grevena) during the baseline period (2011–2014). Ptolemaida and Florina, situated in close proximity to large power plants and open-pit mines, consistently exhibited the highest pollution levels. Mean PM_10_ concentrations in Ptolemaida and Florina were approximately 30–40 μg/m^3^, significantly exceeding those observed in Kozani (mid-20s μg/m^3^) and Grevena (<20 μg/m^3^, *p* < 0.001) during the early 2010s. On average, PM_10_ in Kozani was about 30% lower than in Ptolemaida or Florina, a statistically significant difference [29]. Similar patterns emerged for gaseous pollutants: Florina recorded the highest annual mean NO_2_ (routinely 30–40 ppb), followed by Ptolemaida (~25–30 ppb), whereas Kozani’s NO_2_ levels (~15–20 ppb) were significantly lower (*p* < 0.001) [43]. Florina also demonstrated elevated NO and NO_x_, attributable to emissions from the Meliti plant and municipal heating, with levels substantially exceeding those in Kozani. Although nitrogen oxides (NO_x_) are also emitted by traffic sources, the elevated NO and NO_2_ levels observed in Florina and Ptolemaida primarily reflect emissions from nearby lignite-fired power plants. These facilities are well-documented sources of NO_x_ due to high-temperature combustion processes, and the pronounced spatial gradients observed—markedly higher concentrations in areas adjacent to power plants and substantially lower levels in the rural control area of Grevena—support a dominant industrial rather than traffic-related contribution. Sulfur dioxide was notably elevated in Florina as well—the Florina station registered the highest SO_2_ concentrations among the areas, consistent with the high-sulfur lignite combusted at the Meliti station. Ptolemaida’s SO_2_ was intermediate, and Kozani’s SO_2_ was lowest; all pairwise differences were significant (*p* < 0.001). Collectively, these observations designate Florina and Ptolemaida as high-exposure areas, Kozani as moderate exposure, and Grevena as a low-exposure background site. These comparisons are based on annual mean pollutant concentrations calculated separately for each year (2011–2014), with approximately 12 monthly observations contributing to each annual estimate per monitoring station. Based on these spatial contrasts, Ptolemaida and Florina are classified as high-exposure areas, Kozani as an intermediate-exposure area, and Grevena as a low-exposure background reference site for all subsequent analyses.

Ambient O_3_ exhibited an inverse pattern, tending to be higher in Grevena and Kozani (rural or less polluted locations) and lower in the lignite hubs due to titration by fresh NO emissions. Because ozone levels are less directly tied to local combustion sources, O_3_ was not the primary focus of the health analyses.

Seasonal variation was evident across all locations: concentrations of PM_10_, NO_x_, and SO_2_ increased during winter months (owing to intensified coal heating and more stable atmospheric conditions) and decreased in summer [1]. This seasonality was consistent across years and broadly synchronous throughout the region.

### 3.2. Trends in Pollution from 2011–2014 to 2021

A principal finding is the marked improvement in air quality by 2021 relative to a decade earlier. Between 2014 and 2021, Western Macedonia experienced partial deindustrialization as aging lignite units were retired or operated at reduced capacity. Correspondingly, pollutant levels in 2021 were the lowest on record in all monitored areas. In Kozani, the 2021 annual mean PM_10_ was 18 μg/m^3^, a substantial reduction from the 2011–2014 average of ~28 μg/m^3^ (*p* < 0.001). In Ptolemaida, the 2021 PM_10_ mean declined to ~22 μg/m^3^ from ~35 μg/m^3^ in 2011 (*p* < 0.001) [44]. Florina exhibited a similar decline (2021 mean ~25 μg/m^3^ vs. ~38 μg/m^3^ in 2013, *p* < 0.01). In every location, 2021 PM_10_ concentrations were significantly lower than any year during 2011–2014. The decrease was particularly steep from 2014 to 2021, consistent with decommissioning activities and broader emission reductions. Year-by-year annual mean concentrations are reported to illustrate the temporal evolution of pollutant levels, with the number of underlying monthly observations remaining consistent across years.

Reductions in SO_2_ were even more pronounced. Kozani’s mean SO_2_ in 2021 was nearly 70% lower than a decade earlier (*p* < 0.001), with a similar magnitude of decline in Ptolemaida. Florina—historically the highest for SO_2_—also experienced a marked decrease after 2014, with 2021 levels more than 50% lower than the 2013 peak. Nitrogen oxides likewise diminished: mean NO_2_ in Kozani fell to ~12 ppb in 2021 from ~20 ppb in 2013 (*p* < 0.001), and Ptolemaida’s NO_2_ decreased from ~30 ppb to ~18 ppb (*p* < 0.01). Across areas, 2021 consistently presented the lowest annual NO, NO_2_, and NO_x_ values in the 2011–2021 interval.

The 2021 improvements are attributable largely to diminished lignite combustion emissions. By that year, multiple generating units had been retired or idled, and remaining operations (including a new unit in Ptolemaida) were subject to stricter EU controls. Nationwide COVID-19 restrictions in early 2021 may also have temporarily lowered traffic and industrial activity. Regardless of contributing factors, the evidence confirms substantially reduced community exposure by 2021 relative to the early 2010s.

### 3.3. Hospital Admissions for Cardiovascular Conditions

Across the four hospitals, 16,217 cardiovascular-related admissions were recorded during 2011–2014 and 3785 in 2021 (including all cardiac diagnoses as defined in Methods). Significant inter-hospital differences were evident. Grevena—the least polluted and least populous area—had far fewer annual cardiac hospitalizations than the other areas (typically <200 cardiovascular admissions annually, versus ~600 in Florina and ~1000–1200 in Kozani and Ptolemaida; χ^2^ test for distribution across hospitals, *p* < 0.001). Florina also registered fewer admissions than Kozani or Ptolemaida, consistent with its smaller population, while Kozani and Ptolemaida exhibited the highest and broadly similar case numbers. When adjusted for population, Kozani and Ptolemaida showed the highest rates of cardiovascular hospitalization, whereas Grevena exhibited the lowest. These geographic patterns qualitatively mirror pollution exposure gradients—communities downwind of lignite power plants experienced a greater burden of cardiac hospitalizations than the relatively unpolluted area.

Diagnostic composition further underscored these differences. Table 3 details the distribution of cardiovascular admissions by category (ACS, AF, heart failure, stroke, other) by hospital and year. During baseline years, ACS comprised one of the largest fractions of cardiac admissions in Kozani, Ptolemaida, and Florina, consistent with the substantial burden of coronary artery disease. In 2011, ACS represented approximately 30% of cardiac admissions in Florina Hospital (32.8% precisely). AF was likewise a major contributor, comprising roughly 20–30% of cardiac admissions in various years; at Kozani, AF admissions ranged from ~25–39% during 2011–2013.

Chi-square tests were performed separately for each diagnostic category within each hospital to assess changes in the distribution of cardiovascular admissions across the study years (2011–2014 and 2021). Analyses were based on absolute admission counts (n). Degrees of freedom (df = 4) correspond to the five temporal categories included. Source: author’s own creation.

**Figure 2:**
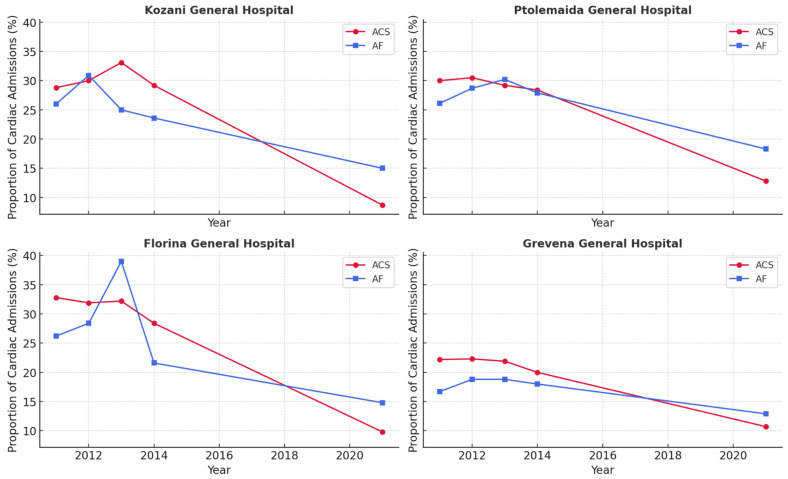
Temporal trends in ACS and AF as percentage of cardiac admissions, comparing the baseline period (2011–2014) with 2021. Source: author’s own creation.

Temporal shifts in diagnostic proportions were prominent. At Kozani General Hospital, the proportions of ACS and AF admissions declined markedly from 2011 to 2021. Chi-square analyses showed significant variation across 2011, 2012, 2013, 2014, and 2021 in three diagnostic categories: ACS (Χ^2^ = 18.05, *p* = 0.001), heart failure (Χ^2^ = 17.72, *p* = 0.001), and AF (Χ^2^ = 13.77, *p* = 0.008). Specifically, ACS decreased from ~28–33% (2011–2013) to <10% (2021), and AF fell from ~26% (2011) to ~15% (2021). In Florina, three diagnostic categories, including ACS, AF, and heart failure, exhibited statistically significant temporal variation. ACS declined from nearly one-third of cardiac admissions in 2011 (32.8%) to 9.8% in 2021. AF accounted for 26.2% of admissions in 2011, rose transiently to ~39% in 2013, and declined to 14.8% in 2021 (Χ^2^ = 10.85, *p* = 0.028). In Ptolemaida, significant temporal changes were observed for ACS, heart failure, and other cardiac diagnoses, whereas AF and stroke did not show statistically significant variation. Overall, the findings indicate a decreasing trend in acute ischemic and arrhythmic events by 2021 compared with earlier years, particularly in Kozani and Florina. Figure 2 visualizes these trends by depicting the percentage of all cardiac admissions attributable to ACS or AF in 2011–2014 versus 2021 for each hospital.

Multiple factors may underlie these temporal patterns. The absolute number of cardiac admissions in 2021 was generally lower than in earlier individual years for most hospitals (Florina recorded 82 total cardiovascular admissions in 2021 versus an annual average of ~130 in 2011–2014). While the COVID-19 pandemic may have influenced absolute hospitalization volumes in 2021, the analysis of diagnostic proportions indicates that the selective decline in ACS and AF admissions cannot be fully explained by healthcare avoidance alone and is more plausibly linked to reduced exposure to pollution-related cardiovascular triggers.

### 3.4. Associations Between Air Pollution and Cardiac Admissions

Associations between monthly pollution levels and monthly cardiovascular admissions were evaluated. Given the multifactorial etiology of cardiac events, modest correlation magnitudes were anticipated; nevertheless, observed associations generally trended positive (higher pollution coinciding with more admissions). A summary table presenting pollutant-specific associations with cardiovascular admissions is provided (Table 4) to facilitate comparison across pollutants and study areas.

Spearman’s rank correlation coefficients (ρ) were calculated between monthly pollutant concentrations and monthly total cardiovascular admissions for each hospital area. All pollutant–region combinations are reported irrespective of statistical significance to ensure transparent presentation of results. Source: author’s own creation.

In Kozani, monthly mean NO_2_ exhibited a weak but statistically significant positive correlation with total cardiac admissions (Spearman ρ = +0.115, *p* = 0.045), indicating that months with higher NO_2_—often a marker of combustion and traffic emissions—tended to coincide with slightly elevated hospitalization counts [45]. In Florina, monthly PM_10_ levels were positively correlated with total cardiac admissions (ρ = +0.138, *p* = 0.017), consistent with higher hospitalization counts during months of worse particulate pollution [40]. In Ptolemaida, the PM_10_ correlation was near the significance threshold (ρ = +0.112, *p* = 0.052), suggesting a similar positive association [46]. In Ptolemaida, SO_2_ showed a weak positive correlation with admissions (ρ = 0.098, *p* = 0.088), although it did not reach statistical significance. Negative correlations were small in magnitude and were observed mainly for O_3_, whereas most combustion-related pollutants showed weak positive correlations across areas. Ozone (O_3_) was included for completeness; however, given its secondary formation and inverse spatial pattern relative to combustion-related pollutants, it was not considered a primary exposure of interest in this study.

The epidemiological analysis demonstrated generally positive associations between ambient pollution metrics and cardiovascular hospital admissions in Western Macedonia, albeit with low correlation coefficients (ρ~0.1). Such magnitudes are expected given potential confounding factors (e.g., meteorology, holidays, healthcare access) and the coarse monthly temporal resolution. Taken together, the spatial contrasts between high- and low-exposure areas and the temporal co-occurrence of declining pollution levels and cardiovascular admissions suggest a consistent directional descriptive association. Although individual pollutant–outcome correlations were modest and did not uniformly reach statistical significance, the observed trends are compatible with improved cardiovascular outcomes following reduced pollution exposure. Although the observational design precludes definitive causal inference, the consistency of spatial gradients, temporal co-variation, and biological plausibility supports a causal-consistent interpretation rather than simple correlation.

## 4. Discussion

This study evaluated the impact of chronic lignite-combustion air pollution on cardiovascular health in Western Macedonia, Greece, and yielded several principal findings. Communities in close proximity to lignite power plants—specifically Ptolemaida and Florina—experienced significantly higher concentrations of PM_10_, NO_x_, and SO_2_, accompanied by a greater burden of cardiovascular hospital admissions than the intermediate-exposure area of Kozani and the low-exposure control area of Grevena. A marked decline in the proportion of cardiovascular hospital admissions attributable to Acute Coronary Syndrome (ACS) and Atrial Fibrillation (AF) was observed between 2011–2014 and 2021. Importantly, the analysis focused on diagnostic proportions rather than absolute admission counts, thereby accounting for differences in overall hospital utilization across years. This approach reduces potential bias related to fluctuations in healthcare-seeking behavior and supports interpretation in terms of changing cardiovascular burden rather than crude incidence. At the hospitals serving Kozani, Ptolemaida, and Florina, the proportions of admissions attributable to ACS and AF fell by half or more by 2021 relative to a decade earlier, mirroring pronounced reductions in particulate and gaseous emissions following partial decommissioning of PPC’s lignite facilities [47]. Short-term correlation analyses further indicated that monthly fluctuations in pollution levels were positively associated with fluctuations in cardiovascular admissions, albeit with modest effect sizes (ρ~0.1–0.14) given the complexity of disease dynamics. Taken together, the contemporaneous improvements in air quality and cardiovascular outcomes support an interpretation consistent with a causal relationship and align with prior environmental epidemiology demonstrating near-term health benefits from pollution reduction [3,48]. It should be noted that 2021 coincided with the COVID-19 pandemic; however, the present analysis intentionally contrasts 2021 with pre-pandemic baseline years (2011–2014) and focuses on proportional diagnostic patterns rather than absolute admission counts, reducing the likelihood that the observed declines in ACS and AF are solely attributable to pandemic-related healthcare disruptions.

These findings accord with an extensive literature linking air pollution to cardiovascular events. Time-series and meta-analytic evidence show that increments in particulate matter are associated with increased myocardial infarction risk; a 10 μg/m^3^ rise in PM_10_ has been associated with ~0.6% higher MI risk in the immediate days following exposure, and pooled estimates for PM_2.5_ suggest an overall relative risk around 1.02 per 10 μg/m^3^ increase for MI hospitalizations [49]. The observed association with NO_2_ in Kozani is consistent with meta-analyses reporting that short-term NO_2_ exposure increases hospital admissions for ischemic heart disease and stroke by a few percent per 10 ppb [50]. Long-term European studies further link chronic exposure to PM and NO_2_ with elevated incidence of coronary events and atherosclerotic progression [51,52]. With respect to AF, the data corroborate growing evidence that air pollution precipitates arrhythmias: short-term PM_2.5_ exposure has been associated with ~1.8% higher AF risk per 10 μg/m^3^, and long-term PM_2.5_ exposure elevates AF incidence in healthy populations [53]. Additional syntheses report significant associations for both particulate and gaseous pollutants with AF prevalence (e.g., OR = 1.03 per 10 ppb NO_2_ short term; OR = 1.07 per 10 μg/m^3^ PM_2.5_ long term), concluding that “all air pollutants exposure had an adverse effect on AF prevalence”. Studies in other contexts—such as urban traffic environments—have shown elevated paroxysmal AF onset following days of high particulate or ozone, including an Italian analysis linking a 50 μg/m^3^ PM_2.5_ increase with nearly doubled AF odds over subsequent days [54,55].

By focusing on a specific source (lignite power plants) and a defined population undergoing energy transition, this study complements broader epidemiology and parallels experiences from “air pollution episodes,” such as the Dublin coal ban and the Chinese Olympic air cleanup, where abrupt air quality improvements yielded measurable cardiovascular benefits [47,48]. In Dublin, restrictions on coal sales produced an approximate 10% reduction in cardiovascular mortality; similarly, the lignite drawdown in Western Macedonia between 2014 and 2021 appears to have reduced acute cardiac events, underscoring that policy-driven emissions cuts can generate tangible public health gains within a few years. Comparisons with other coal regions (e.g., parts of Poland or India) are instructive: pre-2015 PM_10_ levels of ~30–40 μg/m^3^ in Western Macedonia are comparable to or lower than those in some coal belts, where elevated cardiovascular admissions and mortality have been documented near power plants and downwind communities report increased stroke and heart failure hospitalizations [51,52].

The observed associations are biologically plausible. Inhaled particulate matter and co-pollutants provoke pulmonary inflammation and oxidative stress, with systemic cytokine spillover (e.g., IL-6, TNFα) promoting a pro-inflammatory state that destabilizes atherosclerotic plaques, thereby triggering ACS through plaque rupture/erosion and thrombosis [56,57]. Particulate exposure augments coagulability and viscosity (increases in fibrinogen, factor VIII, plasma viscosity), heightening coronary thrombosis risk, while vasoconstriction and transient blood pressure surges—potentially mediated by endothelin-1 and reduced nitric oxide bioavailability—contribute to ischemic events [3,58]. For AF, autonomic imbalance and electrophysiological perturbations are central: pollutants can elicit pulmonary reflexes that increase sympathetic tone and reduce vagal activity, with documented reductions in heart rate variability after exposure. Systemic inflammation and oxidative stress may foster atrial remodeling and fibrosis, and ultrafine particles may directly affect myocardial ion channels, altering refractoriness and promoting triggered activity. Acute pollutant elevations (including ozone) have been linked to immediate increases in AF events, and while the population-attributable fraction for AF due to PM_2.5_ is modest, it is non-negligible at the population level. In Western Macedonia, prolonged high exposure likely accelerated atherosclerosis and contributed to structural cardiac changes (e.g., hypertension-related atrial enlargement), increasing baseline risks of coronary disease and AF; as pollution abated, both chronic progression and acute triggers diminished, aligning with reduced hospitalizations. The AHA statement indicates that while long-term exposure elevates cardiovascular risk, PM_2.5_ reductions are associated with rapid declines in cardiovascular mortality within a few years, implying partial reversibility—consistent with the markedly lower ACS/AF incidence observed in 2021 [3].

The public health and clinical implications are clear. Air quality improvements can yield substantial cardiovascular benefits, and the lignite phase-out in Western Macedonia appears to have delivered measurable reductions in heart attacks and arrhythmias. Emissions-control policies thus function as cardiovascular prevention strategies, supporting international guidance such as the WHO 2021 Air Quality Guidelines, which tightened recommended PM_2.5_ and NO_2_ limits in recognition that even low levels adversely affect health [51,52]. Achieving these targets could translate into reductions in ACS, AF, and other conditions (e.g., heart failure and stroke) also linked to pollution. Clinically, air quality should be incorporated into risk counseling: on high-pollution days, susceptible patients (e.g., those with coronary artery disease, prior MI, heart failure, or AF) may benefit from limiting outdoor exertion, using indoor air filtration, and maintaining optimal medication adherence. Personal-level interventions (N95 masks, indoor air purifiers) show promise in mitigating acute cardiovascular effects, including improvements in ischemic markers and heart rate variability among patients with ischemic heart disease during exposure [59,60].

Several limitations should be acknowledged. The ecological, observational design precludes definitive causal inference, and unmeasured confounding (lifestyle, healthcare access, socioeconomic shifts) cannot be fully excluded. Nonetheless, demographic aging and economic stressors related to mine closures would be expected to increase, not decrease, cardiovascular risk—yet cardiovascular events declined, reinforcing the salience of pollution reduction. Reliance on hospital admissions captures more severe manifestations of ACS/AF and omits milder or out-of-hospital events, potentially underestimating the true burden; diagnostic coding may introduce misclassification, though aggregation under ACS mitigates this concern. Statistical power was limited (approximately 60 monthly observations per site), leaving some near-threshold correlations underpowered; the data gap between 2014 and 2021 constrained continuous interrupted time-series approaches, with 2021 serving effectively as a post-intervention snapshot. Meteorological confounding was not explicitly modeled; while monthly averaging and multi-year coverage dampen some acute confounding, models incorporating temperature and humidity could refine estimates. COVID-19 likely affected both air quality and healthcare-seeking behavior; focusing on diagnostic proportions (ACS/AF shares) reduces, but does not eliminate, such bias.

Despite these caveats, the study has notable strengths. It represents one of the first comprehensive analyses in Greece—and among a limited number globally—to link coal power-plant emissions with specific cardiovascular outcomes using multi-year, real-world data. Inclusion of all major regional hospitals enhances representativeness, and the natural experiment of lignite phase-out strengthens causal inference beyond cross-sectional designs. Employing spatial, temporal, and correlation approaches provides consistent evidence from multiple analytical angles, while Western Macedonia’s pollution profile—dominated by a single industrial source—offers clearer attribution than complex urban settings. A further strength is the explicit inclusion of AF, an outcome often underexamined in environmental cardiology despite substantial morbidity and rising global incidence [53]. Demonstrating associations between pollution and AF in this setting contributes timely evidence to an emerging field and supports calls to recognize pollution as an arrhythmic trigger, reinforcing the argument that air-quality interventions may curb the growing AF burden alongside reductions in ischemic events [61].

## 5. Conclusions

Exposure to air pollution from lignite combustion is associated with a higher hospital burden of acute cardiovascular events, notably Acute Coronary Syndromes (ACS) and Atrial Fibrillation (AF), in Western Macedonia. The evidence presented indicates that prolonged exposure to elevated PM_10_, NO_x_, and SO_2_ emissions corresponded with higher proportions of cardiovascular hospital admissions due to ACS and AF, while the substantial reduction in these emissions by 2021 was accompanied by a pronounced decline in the relative contribution of these conditions to overall cardiac admissions. In other words, improved air quality were accompanied by improved cardiovascular outcomes within this population. These findings remain valid irrespective of the modest short-term correlation coefficients observed in monthly analyses, as the overarching temporal trends and their consistency with established scientific literature collectively support a causal interpretation.

From a medical and public health perspective, these findings reaffirm that air pollution represents a major modifiable risk factor for cardiovascular disease. Mitigating ambient pollution—through transitioning from coal to cleaner energy sources, enforcing emission control technologies, and adhering to international air quality standards—should be considered a cornerstone of cardiovascular prevention strategies. Clinicians operating in polluted regions should acknowledge days of poor air quality as periods of elevated cardiovascular risk for vulnerable patients and provide appropriate preventive guidance. Public health authorities should likewise issue timely advisories and ensure that local populations are aware of exposure-reduction measures. The experience of Western Macedonia provides evidence consistent with the idea that communities achieving cleaner air can expect measurable decreases in heart attacks, arrhythmias, and related hospital burdens within a relatively short time frame.

The “natural experiment” of Western Macedonia’s de-lignification offers convergent empirical evidence: when the air cleared, fewer patients filled the cardiac wards. This regional transition serves as a microcosm of what can be achieved globally through sustained emission reductions. The results underscore the importance of maintaining and expanding air quality improvement initiatives in Greece and beyond, emphasizing that clean air is not only an environmental imperative but also a powerful determinant of public health.

Future research should continue to build on these findings by extending monitoring to longer-term cardiovascular outcomes, such as stroke incidence, chronic heart failure, and overall cardiovascular mortality, as the region’s air quality continues to evolve. Investigations integrating individual-level exposure data, meteorological variables, and personal health risk factors would further refine causal understanding. Additionally, comparative studies across other former coal regions in Europe could elucidate the generalizability of these outcomes and quantify the broader public health benefits of energy transitions. Ultimately, sustained interdisciplinary research linking environmental science, clinical cardiology, and policy evaluation will be essential for guiding effective interventions and ensuring that the health benefits of cleaner air are fully realized and equitably distributed.

## Figures and Tables

**Table 1:** Geographic coordinates of air quality monitoring stations and hospitals included in the study.

Facility Type	Name	Municipality/Area	Latitude (°N)	Longitude (°E)
Monitoring station	Filotas	Florina	40.781	21.610
Monitoring station	Koilada	Kozani	40.294	21.798
Monitoring station	Kato Komi	Kozani	40.236	21.784
Monitoring station	Amyntaio	Florina	40.689	21.679
Monitoring station	Florina	Florina	40.781	21.409
Monitoring station	Agios Dimitrios	Kozani	40.496	21.803
Monitoring station	Meliti	Florina	40.878	21.584
Monitoring station	Pontokomi	Kozani	40.350	21.733
Monitoring station	Anargyroi	Florina	40.671	21.702
Monitoring station	Grevena	Grevena	40.084	21.427
Hospital	General Hospital of Kozani	Kozani	40.300	21.788
Hospital	General Hospital of Ptolemaida	Ptolemaida	40.514	21.679
Hospital	General Hospital of Florina	Florina	40.781	21.409
Hospital	General Hospital of Grevena	Grevena	40.083	21.427

Source: author’s own creation.

**Table 2:** Mean concentrations (±SD) of major air pollutants in Western Macedonia (2011–2014).

Pollutant	Grevena (Low Exposure, Control)	Kozani (Moderate Exposure)	Ptolemaida (High Exposure)	Florina (High Exposure)
PM_10_ (μg/m^3^)	18.5 ± 6.7 ^c^	25.6 ± 7.8 ^a^	38.9 ± 10.5 ^b^	40.3 ± 11.2 ^b^
SO_2_ (μg/m^3^)	4.0 ± 2.5 ^a^	5.2 ± 3.4 ^a^	14.7 ± 5.8 ^b^	23.8 ± 6.5 ^c^
NO (ppb)	3.8 ± 1.9 ^a^	4.5 ± 2.2 ^a^	7.8 ± 3.3 ^b^	11.5 ± 4.7 ^c^
NO_2_ (ppb)	12.8 ± 3.9 ^a^	17.2 ± 4.8 ^a^	27.5 ± 6.3 ^b^	35.4 ± 8.1 ^c^
NO_x_ (ppb)	15.6 ± 5.1 ^a^	22.4 ± 5.9 ^a^	35.1 ± 7.8 ^b^	45.8 ± 9.3 ^c^
O_3_ (ppb)	38.6 ± 6.9 ^c^	32.7 ± 6.2 ^b^	25.9 ± 5.7 ^a^	24.3 ± 5.5 ^a^

PM_10_ = particulate matter ≤ 10 μm; SO_2_ = sulfur dioxide; NO = nitric oxide; NO_2_ = nitrogen dioxide; NO_x_ = total nitrogen oxides (NO + NO_2_). Grevena represents a low-exposure reference site with minimal influence from lignite power generation. Superscript letters (a, b, c) denote groups whose mean values differ significantly (*p* < 0.05); identical letters indicate no statistically significant difference. Source: author’s own creation.

**Table 3:** Temporal variation in cardiovascular admission categories across hospitals in Western Macedonia (2011–2014 vs. 2021).

Hospital	Diagnostic Category	χ^2^	df	*p*-Value
Kozani	Acute Coronary Syndrome (ACS)	18.05	4	0.001
	Atrial Fibrillation (AF)	13.77	4	0.008
	Heart Failure	17.72	4	0.001
	Stroke	4.62	4	0.329
	Other cardiac diagnoses	3.91	4	0.418
Ptolemaida	Acute Coronary Syndrome (ACS)	11.26	4	0.024
	Atrial Fibrillation (AF)	6.14	4	0.189
	Heart Failure	10.87	4	0.028
	Stroke	3.48	4	0.482
	Other cardiac diagnoses	9.96	4	0.041
Florina	Acute Coronary Syndrome (ACS)	10.78	4	0.029
	Atrial Fibrillation (AF)	10.85	4	0.028
	Heart Failure	9.75	4	0.045
	Stroke	5.02	4	0.285
	Other cardiac diagnoses	8.67	4	0.070
Grevena	Acute Coronary Syndrome (ACS)	3.12	4	0.538
	Atrial Fibrillation (AF)	2.74	4	0.602
	Heart Failure	3.46	4	0.485
	Stroke	4.08	4	0.395
	Other cardiac diagnoses	5.11	4	0.276

**Table 4:** Spearman correlations between monthly air pollutant concentrations and total cardiovascular hospital admissions (2011–2014, 2021).

Area	Pollutant	Spearman ρ	*p*-Value
Kozani	PM_10_	0.041	0.712
	SO_2_	0.067	0.548
	NO	0.052	0.641
	NO_2_	**0.115**	**0.045**
	NO_X_	0.081	0.402
	O_3_	−0.029	0.781
Ptolemaida	PM_10_	**0.112**	**0.052**
	SO_2_	0.098	0.088
	NO	0.073	0.312
	NO_2_	0.084	0.221
	NO_X_	0.091	0.176
	O_3_	−0.046	0.604
Florina	PM_10_	**0.138**	**0.017**
	SO_2_	0.094	0.101
	NO	0.109	0.061
	NO_2_	0.102	0.079
	NO_X_	0.117	0.054
	O_3_	−0.062	0.421
Grevena	PM_10_	0.028	0.816
	SO_2_	0.031	0.792
	NO	0.019	0.881
	NO_2_	0.024	0.847
	NO_X_	0.036	0.741
	O_3_	−0.041	0.683

## Data Availability

The original contributions presented in this study are included in the article. Further inquiries can be directed to the corresponding author.

## Abbreviations

The following abbreviations are used in this manuscript:

ACS	Acute Coronary Syndromes
AF	Atrial Fibrillation
PPC	Public Power Corporation
